# Ecological traits of the world’s primates

**DOI:** 10.1038/s41597-019-0059-9

**Published:** 2019-05-13

**Authors:** Carmen Galán-Acedo, Víctor Arroyo-Rodríguez, Ellen Andresen, Ricard Arasa-Gisbert

**Affiliations:** Instituto de Investigaciones en Ecosistemas y Sustentabilidad, Universidad Nacional Autónoma de México, Antigua Carretera a Pátzcuaro no. 8701. Ex-Hacienda de San José de la Huerta, C.P. 58190, Morelia, Michoacán Mexico

**Keywords:** Ecosystem ecology, Conservation biology, Tropical ecology, Ecological networks

## Abstract

Ecosystems largely depend, for both their functioning and their ecological integrity, on the ecological traits of the species that inhabit them. Non-human primates have a wide geographic distribution and play vital roles in ecosystem structure, function, and resilience. However, there is no comprehensive and updated compilation of information on ecological traits of all the world’s primate species to accurately assess such roles at a global scale. Here we present a database on some important ecological traits of the world’s primates (504 species), including home range size, locomotion type, diel activity, trophic guild, body mass, habitat type, current conservation status, population trend, and geographic realm. We compiled this information through a careful review of 1,216 studies published between 1941 and 2018, resulting in a comprehensive, easily accessible and user-friendly database. This database has broad applicability in primatological studies, and can potentially be used to address many research questions at all spatial scales, from local to global.

## Abstract (summary)

**Table:** 

Design Type(s)	species comparison design • data integration objective • biodiversity assessment objective
Measurement Type(s)	Ecology
Technology Type(s)	digital curation
Factor Type(s)	geographic location • Species
Sample Characteristic(s)	Primates

Machine-accessible metadata file describing the reported data (ISA-Tab format)

## Background & Summary

The ecological traits of species determine their contribution to ecosystem properties and their tolerance to environmental changes, including human-induced disturbances^1,2^. Non-human primates show a large variation in ecological traits (e.g., body mass varies from 0.03 to 130 kg)^3^. They play key roles in the structuring and functioning of the ecosystems where they occur, acting as herbivores, seed dispersers, and predators^3–5^. Primates have a wide distribution, inhabiting a great variety of the Earth’s ecosystems, in both tropical and temperate latitudes^3^. However, to our knowledge, very few studies have assessed the ecological roles of primates at a global scale (but see^6,7^), probably due to the lack of a global database of ecological traits. Also, despite the current conservation crisis of the world’s primates^4^, there is little information on the ecological traits that can make primate species more prone to extinction in human-modified landscapes^8–10^. Because primates inhabit many of the most diverse and threatened ecosystems in the world^3^, understanding the relationships between the ecological traits of species and their responses to habitat disturbance is of key relevance. This information is not only needed for primate conservation, but also to preserve the many other species of organisms with which primates interact and thus the ecological processes in which they are involved^4^.

**Fig. 1:**
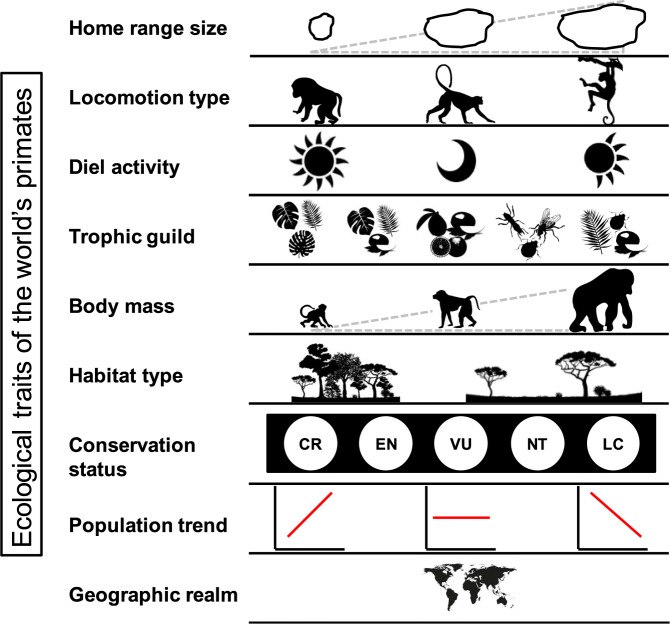
Summary of the ecological traits of the world’s primates included in the database. From left to right pictures represent: (1) home range size gradient from small to large; (2) locomotion types are terrestrial, both locomotion types, and arboreal; (3) diel activity includes diurnal, nocturnal and cathemeral; (4) trophic guild includes folivore, folivore-frugivore, frugivore, insectivore, omnivore, and gummivore (the latter not depicted); (5) body mass gradient from small to large; (6) habitat type includes seven categories (see text) but only two are depicted as examples (forest and savannah); (7) IUCN conservation status includes seven categories, with five depicted here (CR critically endangered, EN endangered, VU vulnerable, NT near threatened and LC least concern); (8) population trend is represented by three graphs indicating increasing, stable and decreasing populations; and (9) geographic realm is represented by a global map. Images used with permission from Microsoft.

Despite some efforts to compile ecological information on primate species, available databases are usually restricted to specific geographic regions (e.g., Madagascar^11,12^), are not up-to-date with recent information^13^ or do not include details on the methods (e.g., sampling effort) used for obtaining the data^14^. Also, information is widely scattered in different types of sources, including hard-to-access publications^3^, and user-restricted web pages (e.g., www.alltheworldsprimates.com). Furthermore, most resources include large amounts of information for some species, making it difficult to find specific ecological traits for many species. Thus, our main objective is to provide for the scientific community a comprehensive, easily accessible and user-friendly database of some traits with ecological and conservation significance for the world’s primates (Fig. 1). The database includes information on primates’ home range size (365 out of 504 species), locomotion type (497 species), diel activity (504 species), trophic guild (425 species), body mass (462 species), habitat type (480 species), current conservation status (504 species), population trend (393 species) and geographic realm (504 species). The structure of the database allows for different levels of organization (e.g., by taxon and trait).

Potential uses of this database include the assessment of (1) the functional structure of primate communities, (2) the influence of primate species on ecosystem function and services, (3) the functional signal of species’ responses to habitat disturbances across ecosystems, (4) the relationship between primate conservation efforts and ecosystem conservation, (5) the relationships between diversity metrics and other ecosystem attributes, including function and resilience, and (6) the ecological roles of primates at different spatial scales. For example, we have used this database to test, at a global scale, which ecological traits of primates correlate more strongly with the use of the anthropogenic matrix in human-modified landscapes^10^.

## Methods

Ecological traits included in the database are: home range size, locomotion type, diel activity, trophic guild, body mass, habitat type, current conservation status, population trend, and geographic realm. We selected these variables because of their well-known ecological significance^15^. For instance, body mass is correlated with many life-history traits that can affect the structure and dynamics of ecological networks^16,17^ and is recognized as a variable that can have profound impact across multiple scales of organization, from the individual to the ecosystem level^16,18–20^. We updated the primate taxonomic nomenclature following Estrada *et al*.^4^, which is mostly based on the International Union for Conservation of Nature (IUCN). Although we also indicate the most up-to-date primate taxonomic nomenclature published in the Integrated Taxonomic Information System (http://www.itis.gov/) of the Order Primates, we have probably not noticed the last taxonomic changes for some species. Thus, we kindly request users to contact the corresponding author if they find any error in the database to maintain it as updated as possible.

We collected data from 1,216 studies published between 1941 and 2018, including scientific articles, books, reports, dissertations, and web pages. The literature search included publications in English, Spanish, French, German and Portuguese. Geographic realm was extracted from Mittermeier *et al*.^3^ Current conservation status, population trend and habitat type of each species were obtained from the IUCN database, using the ‘letsR’^21^ package for R, version 3.0.1.^22^. The IUCN defines habitat type as “the major habitats in which each taxon occurs” https://www.iucnredlist.org/resources/habitat-classification-scheme. When there was no information on these variables for a given primate species in the IUCN database, we used information available in other sources by actively searching in the World Wide Web (public domain and scientific publications). When different sources yielded different information for a given species and trait, we recorded this information in different rows. This procedure allowed us to have a more comprehensive, accurate and objective database.

Information on home range sizes is given in hectares. When information and primary sources are available for the home range data and a study reports more than one home range size, we average all the values. We also include the minimum and maximum values, the number of groups and mean group size, the method used (e.g., minimum convex polygon), alternative home range and alternative method (when available), and the study duration. Locomotion type refers to the main way in which an animal moves in its environment. Arboreal locomotion type includes primate species that are strictly arboreal, which in undisturbed forest very rarely go to the ground; terrestrial type includes primate species that are mainly terrestrial, i.e., carrying out most of their daily activity on the ground; the locomotion category ‘both’ includes primate species which are commonly active on both substrates, ground as well as trees. Diel activity is categorized as diurnal (i.e., main behavioral activities occurring at daytime), nocturnal (i.e., main behavioral activities occurring at nighttime) and cathemeral (i.e., behavior occurring both at day and nighttime). In terms of trophic guild we consider six general groups: frugivore (>60% of fruits/seeds in diet), folivore (>60% leaves in diet), folivore-frugivore (diet comprised of both fruits/seeds and leaves in similar proportions), omnivore (diet comprised of both plants and animals in similar proportions), insectivore (>50% of arthropods in diet) and gummivore (diet dominated by plant exudates). We include in the trophic guild data base the percentage of fruit, leaves, flowers, seeds, animal matter, nectar and other, when available. We also include, when available, the study region, data type (e.g., feeding records, feeding time), study duration, group size and other general comments (e.g., when fruits and seeds are pooled). Body mass is expressed in kilograms; values for this variable can represent reported individual values, reported averages, or calculated averages (when a study included more than one body mass datum). When available, we separately report the mean body mass of adult males and females. Regarding habitat type, we include seven categories of major natural habitats in which a species occurs: (1) forest, includes ecosystems such as tropical wet forest, cloud forest, dry forest, montane forest, temperate forest and semideciduous forest; (2) savannah, includes savannah forest and savannah mosaics; (3) shrubland, includes ecosystems dominated by shrubs, such as scrub, brush and bush; (4) grassland, includes ecosystems mainly composed of grasses and other herbaceous plants; (5) wetlands, includes ecosystems such as swamps, flooded forest, swampy forest, wetlands and mangroves; (6) rocky areas, includes ecosystems such as inland cliffs and mountain peaks; and, (7) desert.

IUCN threat categories include Critically Endangered, Endangered, Vulnerable, Near Threatened, Least Concern, Data Deficient and Not Evaluated. Population trend includes increasing, stable and decreasing populations. For these two variables we include the year of evaluation for each entry. Finally, we consider four main geographic realms: Asia, Mainland Africa, Madagascar and Neotropics. African primates were classified in two groups, Mainland Africa and Madagascar, because these two land masses span the distribution of two highly divergent primate suborders (catarrhines and strepsirrhines, respectively). These methods are expanded versions of descriptions used in our recently published study^10^. Data files are stored in Zenodo^23^.

## Data Records

The complete database for the ecological traits of primates consists of seven different data files with descriptive names (Table 1). Data files are stored in Zenodo^23^. We also include a text file “References.txt” that contains all the references included as numbers in the data files. The first row of each data file is the header containing the variables’ names; each of the following rows presents data for a single primate species and a single information source. A given species can appear in more than one row because in some cases we included ecological traits from more than one source.

**Table 1:** Summary information for the seven data files comprising the database of ecological traits for the world’s primates.

Data file name	N species	Abbreviations	N rows	N columns	File size
HomeRange.csv	365	NA = No information	750	15	89 KB
		NI = Primary source not available in the internet for data extraction, or data not reported by the study			
Locomotion.csv	497	NA = No information	553	7	49 KB
		AR = Arboreal			
		T = Terrestrial			
		BOTH = Arboreal and terrestrial locomotion			
DielActivity.csv	504	D = Diurnal	528	7	46 KB
		N = Nocturnal			
		CA = Cathemeral			
TrophicGuild.csv	425	NA = No information	571	20	103 KB
		NI = Primary source not available in the internet for data extraction, or data not reported by the study			
BodyMass.csv	462	NA = No information	630	9	62 KB
Habitat.csv	480	NA = No information	527	14	54 KB
IUCN_Poptrend_Realm.csv	504	NE = Not evaluated	527	11	57 KB
		DD = Data deficient			
		LC = Least concern			
		NT = Near threatened			
		VU = Vulnerable			
		EN = Endangered			
		CR = Critically endangered			
		NA = No information			
		I = Increasing			
		D = Decreasing			
		S = Stable			
		M_Africa = Mainland Africa			

## Technical Validation

Most of the records included in the database are based on published material in peer-reviewed scientific journals and books, and thus we have confidence in their accuracy. Also, for each specific datum we include the corresponding reference in the database, allowing users to both assess the validity and consult the original sources. Moreover, we have carefully checked the database for possible redundancies and errors. When a specific datum was considered non-reliable (e.g., very extreme or contradictory values, and values obtained with questionable methodology) we did not include it in the database. We also used the ‘validate’^24^ R package to check the database for structural integrity (i.e., its internal organization). Our aim is to keep updating data for the traits recorded and incorporate other traits such as social attributes and other physical measures in the future. Data will be corrected and updated if any errors or updates are reported to the corresponding author.

## Usage Notes

We would appreciate if researchers cite the database stored in Zenodo^23^ in the specific version used, as well as this publication, when using all or part of the database.

## ISA-Tab metadata file

Download metadata file

## Data Availability

Code for the technical validation can be found archived in the Zenodo repository as “Test_validation”^23^.
